# CXCR7 antagonism prevents axonal injury during experimental autoimmune encephalomyelitis as revealed by *in vivo *axial diffusivity

**DOI:** 10.1186/1742-2094-8-170

**Published:** 2011-12-06

**Authors:** Lillian Cruz-Orengo, Ying-Jr Chen, Joong Hee Kim, Denise Dorsey, Sheng-Kwei Song, Robyn S Klein

**Affiliations:** 1 Department of Internal Medicine, Washington University School of Medicine, 660 S. Euclid Ave, St. Louis, MO, 63110, USA; 2 Department of Radiology, Washington University School of Medicine, 660 S. Euclid Ave, St. Louis, MO, 63110, USA; 3 Department of Anatomy and Neurobiology, Washington University School of Medicine, 660 S. Euclid Ave, St. Louis, MO, 63110, USA; 4 Department of Pathology and Immunology, Washington University School of Medicine, 660 S. Euclid Ave, St. Louis, MO, 63110, USA

**Keywords:** chemokine, EAE, axon, DTI, T cell, multiple sclerosis

## Abstract

**Background:**

Multiple Sclerosis (MS) is characterized by the pathological trafficking of leukocytes into the central nervous system (CNS). Using the murine MS model, experimental autoimmune encephalomyelitis (EAE), we previously demonstrated that antagonism of the chemokine receptor CXCR7 blocks endothelial cell sequestration of CXCL12, thereby enhancing the abluminal localization of CXCR4-expressing leukocytes. CXCR7 antagonism led to decreased parenchymal entry of leukocytes and amelioration of ongoing disease during EAE. Of note, animals that received high doses of CXCR7 antagonist recovered to baseline function, as assessed by standard clinical scoring. Because functional recovery reflects axonal integrity, we utilized diffusion tensor imaging (DTI) to evaluate axonal injury in CXCR7 antagonist- versus vehicle-treated mice after recovery from EAE.

**Methods:**

C57BL6/J mice underwent adoptive transfer of MOG-reactive Th1 cells and were treated daily with either CXCR7 antagonist or vehicle for 28 days; and then evaluated by DTI to assess for axonal injury. After imaging, spinal cords underwent histological analysis of myelin and oligodendrocytes via staining with luxol fast blue (LFB), and immunofluorescence for myelin basic protein (MBP) and glutathione S-transferase-π (GST-π). Detection of non-phosphorylated neurofilament H (NH-F) was also performed to detect injured axons. Statistical analysis for EAE scores, DTI parameters and non-phosphorylated NH-F immunofluorescence were done by ANOVA followed by Bonferroni post-hoc test. For all statistical analysis a p < 0.05 was considered significant.

**Results:**

*In vivo *DTI maps of spinal cord ventrolateral white matter (VLWM) axial diffusivities of naïve and CXCR7 antagonist-treated mice were indistinguishable, while vehicle-treated animals exhibited decreased axial diffusivities. Quantitative differences in injured axons, as assessed via detection of non-phosphorylated NH-F, were consistent with axial diffusivity measurements. Overall, qualitative myelin content and presence of oligodendrocytes were similar in all treatment groups, as expected by their radial diffusivity values. Quantitative assessment of persistent inflammatory infiltrates revealed significant decreases within the parenchyma of CXCR7 antagonist-treated mice versus controls.

**Conclusions:**

These data suggest that CXCR7 antagonism not only prevents persistent inflammation but also preserves axonal integrity. Thus, targeting CXCR7 modifies both disease severity and recovery during EAE, suggesting a role for this molecule in both phases of disease.

## Background

Axonal injury is a critical factor in the progression of neurologic deficits in patients with multiple sclerosis (MS) [1]. Axonal degeneration may occur as a result of oligodendrocyte death and demyelination due to alterations in trophic support and/or impaired mitochondrial bioenergetics [2-7]. Recent studies also indicate that infiltrating leukocytes may directly induce axonal damage that is reversible and occurs in the absence of demyelination [8]. Disease modifying therapies that limit the formation and extent of inflammatory lesions may therefore provide the best approach for preventing disability. In addition, imaging modalities that identify injured axons are critical for monitoring patient responses to these agents.

Recent data examining the dynamic expression of the chemokine CXCL12 at the blood-brain barrier (BBB) indicate that activity of CXCR7, a CXCL12 receptor that sequesters the chemokine intracellularly [9-11], is critical for the entry of infiltrating leukocytes in mice with experimental autoimmune encephalomyelitis (EAE), a murine model for MS [12]. CXCL12 expression along abluminal surfaces of the CNS vasculature normally localizes infiltrating CXCR4-expressing leukocytes to perivascular spaces, thereby restricting their entry into the CNS [13,14]. Loss of abluminal CXCL12, which is specific to MS [15], occurs via cytokine-mediated, up-regulation of CXCR7 by CNS endothelial cells [12]. Administration of a specific CXCR7 antagonist led to retention of abluminal CXCL12 expression at the BBB microvasculature, preventing the pathological entry of immune cells into the CNS parenchyma. Animals that received CXCR7 antagonist exhibited a dose-dependent decrease in peak disease severity and amelioration of ongoing disease [12]. In all circumstances, high doses of CXCR7 antagonist also led to complete clinical recovery while vehicle or untreated animals exhibited chronic deficits. The lack of detectable clinical deficits in animals with limited parenchymal entry of immune cells supports the notion that inflammation leads to reversible axonal injury, which can be ameliorated by targeting CXCR7.

Quantitative analysis of white matter injury in models of MS has been fraught with difficulties due to uneven qualities of the pathology. Diffusion Tensor Imaging (DTI) has emerged as a powerful and sensitive tool to analyze white matter disease. Specifically, DTI has been utilized to assess axonal damage and demyelination *in vivo *in both MS and in animal models such as EAE [16-22]. DTI measures the directional diffusivities of water molecules, which reflect the microstructural organization in biological specimens [23,24]. Radial diffusivity, the diffusion of water perpendicular to the axonal fiber, is associated with myelin integrity and therefore increases during demyelination [22,25]. Axial diffusivity, which reflects microscopic water movement parallel to the axonal fiber, is decreased with loss of axonal integrity [16,17,20,22]. DTI is therefore suitable for the quantitative assessment of myelin versus axonal injury in preclinical studies evaluating novel targets for the treatment of MS.

In the current study, we utilized *in vivo *DTI to examine axonal and myelin injury in vehicle- versus CXCR7 antagonist-treated mice after recovery from EAE. We observed that axial diffusivity within the ventrolateral white matter (VLWM) of mice treated with high doses of CXCR7 antagonist was comparable to naïve mice whereas untreated or vehicle-treated animals showed significant axial diffusivity reduction, suggesting axonal damage. However, we did not observe changes in radial diffusivity between treatment groups, suggesting that CXCR7 antagonism did not impact on levels of myelin during recovery. These results validate the relevance of CXCR7 as a disease modifying molecule not only during the effector phase of EAE, by preventing intraparenchymal leukocyte migration, but also during the recovery phase, by preserving axonal integrity. Moreover, the results also support the use of DTI for assessing the *in vivo *therapeutic efficacy of treatments for CNS autoimmune diseases.

## Materials and methods

### Animals and antibodies

C57BL/6 mice (Jackson Labs, Bar Harbor, ME) were maintained in pathogen free conditions (Department of Comparative Medicine, Washington University, St. Louis, MO) and studies were performed in compliance with the guidelines of the Washington University School of Medicine Animal Studies Committee. Antibodies utilized include rabbit anti-CD3 (Dako, Glostrup, Denmark), rat anti-GFAP (Invitrogen, Carlsbad, CA), rabbit anti-GST-π (Assays Designs-Enzo Life Sciences, Inc. Farmingdale, NY), rat anti-MBP (Abcam, Cambridge, MA) and mouse monoclonal SMI-32 (Covance, Emeryville, CA). IgG from rabbit, rat (Invitrogen, Carlsbad, CA) and mouse (BD Pharmigen, San Diego, CA) were used as isotype controls. Secondary detection was done using goat anti-rat, anti-rabbit and anti-mouse conjugated to Alexa-555 and goat anti-rabbit, and anti-rat conjugated to Alexa-488 (Molecular Probes-Invitrogen, Carlsbad, CA).

### Experimental autoimmune encephalomyelitis induction

Autoreactive Th1 cells directed to myelin oligodendrocyte glycoprotein peptide (MOGp) were generated as previously described [12]. Briefly, naïve C57BL/6 mice were immunized with 50 μg msMOGp35-55 (GenScript, Piscataway, NJ) and 50 μg *Mycobacterium tuberculosis *H37Ra peptide emulsified in Freund's adjuvant (both from Difco Laboratories, Detroit, MI). After 14 days, polarized Th1 cells were harvested from spleen using a nylon wool column. MOG-reactive Th1 cells were restimulated and expanded in culture according to standard protocols prior to adoptive transfer to naïve recipients [12]. 5 × 10^6 ^Th1 cells were incubated with 25 × 10^6 ^stimulators-cells obtained from naïve splenocytes. Incubation proceeded in the presence of MOG, anti-IL-4, IL-2 (both generated in the lab) and IL-12 (BD Pharmigen, San Diego, CA) in RPMI enriched media. Cells were incubated at 37°C for 7 days. After separation with Histopaque (Sigma Aldrich, Saint Louis, MO) 1 × 10^7 ^Th1 cells were restimulated with 5 × 10^7 ^stimulators-cells for other 7 days as described above. Th1 cells underwent a third stimulation of only 4 days in the presence of MOG and IL-2. Following Histopaque separation cells were resuspended in HBSS at 1 × 10^7 ^MOG-reactive Th1 cells/300 ul per mouse ratio. Adoptive transfer of 10 × 10^6 ^MOG-reactive Th1 cells per mouse was done retro-orbitally. Recipient mice were monitored for clinical manifestations of EAE by following their body weight and graded for disease with the following score system: 1, tail weakness; 2, difficulty righting; 3, hind limb paralysis; 4, forelimb weakness; 5, moribund or dead.

### In vivo administration of CCX771, a CXCR7 antagonist

Mice underwent daily subcutaneous injection of either vehicle (10% Captisol) or CCX771 (ChemoCentryx, Mountainview, CA) at doses of 5 or 10 mg/kg of body weight beginning 12 hours after adoptive transfer (5 mice per treatment group). A cohort of mice started with vehicle and changed to CCX771 at a dose of 10 mg/kg of body weight when achieved a score of 1. Dosing with CXCR7 antagonist or vehicle and monitoring for clinical progression continued for 28 days.

### Diffusion Tensor Imaging (DTI) and analysis

Twenty five EAE mice and five naïve control littermates underwent *in vivo *DTI with isoflurane/oxygen anesthesia (5% induction and 1% maintenance) delivered by a custom nose cone that also allowed respiratory-gated acquisition. The mice were placed in a custom holder designed to immobilize the spine and isolate respiratory motion. An actively detuned radiofrequency transmit coil (6 cm internal diameter × 10 cm length) was used with a receiver coil (16 mm internal diameter × 9 mm length) designed to fit around the spine of the mouse. The entire preparation was placed in an Oxford Instruments 200/330 magnet (4.7 T, 40 cm clear bore) equipped with a 20 cm inner-diameter, actively shielded Magnex gradient coil (up to 60 G/cm, 280 μs rise time). Core temperature was maintained at 37°C with circulating warm water. The magnet, gradient coil, and gradient power supply were interfaced with a INOVA console (Varian NMR Systems, Palo Alto, CA) controlled by a Sun Blade 1500 workstation (Sun Microsystems, Santa Clara, CA).

Axial scout images of the spine were acquired for proper localization of spinal cord level. Multiple transverse slices covering spinal cord lumbar enlargement were obtained using a Stejskal-Tanner [16,17,20,26] spin-echo diffusion weighted sequence with the following acquisition parameters: TR ~1500 msec. (determined by the respiratory rate of the mouse), TE of 37 msec., number of excitations equals 2, slice thickness of 1 mm, spinal cord field of view of 1 cm × 1 cm, data matrix of 128 × 128 (zero-filled to 256 × 256). Diffusion-sensitizing gradients were applied in six orientations: (Gx, Gy, Gz) equal to (1, 1, 0), (1, 0, 1), (0, 1, 1), (-1, 1, 0), (0, -1, 1), and (1, 0, -1) with a gradient strength of 9 G/cm, duration (*δ*) equal to 7 msec., and separation (Δ) of 18 msec., to obtain *b *values of 0 and 0.750 s/mm^2^. Acquisition time was approximately one hour for each spinal cord scanning session.

Using a weighted linear least square method, diffusion tensors and DTI parameters were generated. In this study, three DTI parameter maps were used, relative anisotropy (RA), axial diffusivity (λ||), and radial diffusivity (λ⊥) [27]. Regions of interest (ROIs) encompassing the ventrolateral white matter (VLWM) were drawn manually on relative anisotropy (RA) maps projecting to axial and radial diffusivity maps to assess axon and myelin integrity respectively using ImageJ v1.37 software (developed at the U.S. National Institutes of Health and available on the Internet at http://rsbweb.nih.gov/ij/)[28-30]. Injured VLWM region was identified using the baseline axial diffusivity threshold derived from the spinal cord of naïve mice.

### Histological and immunofluorescent analyses

Murine spinal cords were isolated for histological analysis after imaging. Briefly, anesthetized animals were intracardially perfused with PBS and fixed in 4% PFA followed by overnight post-fixation in 4% PFA. Spinal cords were cryoprotected in 30% sucrose prior to embedding in OCT media for cryosectioning. For lumbar segment identification luxol fast blue (LFB) was done following standard procedure and visualized on the Zeiss Axioskop MOT Fluorescent microscope and AxioVision software (both from Carl Zeiss International, Jena, Germany).

Immunodetection of CD3+ lymphocytes within CNS parenchyma was performed via co-labeling with GFAP. Sections from lumbar segments of the spinal cord were permeabilized and blocked in 0.1% Triton X-100 and 10% goat serum for 60 minutes at room temperature. Sections were incubated with primary antibody overnight at 4°C, washed in PBS, incubated with secondary antibodies for 60 minutes at room temperature and counterstained with ToPro-3 (Invitrogen, Carlsbad, CA) to detect nuclei. Immunostained sections were visualized on the Zeiss LSM 510 META Confocal Laser Scanning Microscope (Carl Zeiss International, Jena, Germany). Measurement of CD3+ pixels within the ROIs encompassing parenchyma and meninges was done using the public domain NIH Image program ImageJ.

To facilitate immunodetection of myelin basic protein (MBP) and glutathione S-transferase-π (GST-π) frozen sections from lumbar segments L2/L3 were submitted to antigen retrieval in 0.1% trypsin, 0.1% CaCl in 0.05 M Tris pH 7.4 at 37°C for 10 min. Then sections were permeabilized and blocked in 0.1% Triton X-100 and 3% goat serum for 60 minutes at room temperature. Primary antibody was incubated overnight at 4°C. Slides were washed in 0.2% fish skin gelatin in PBS followed by incubation of secondary antibodies for 60 minutes at room temperature. After washing in 0.2% fish skin gelatin in PBS, slides were nuclear stained with DAPI (Invitrogen, Carlsbad, CA) and coverslip with Vectashield (Vector Laboratories, Inc. Burlingame, CA) before being visualized on the Zeiss Axioskop MOT Fluorescent microscope and AxioVision software (both from Carl Zeiss International, Jena, Germany).

For immunodetection of neurofiliament H frozen sections from lumbar segments L2/L3 were permeabilized and blocked in 0.1% Triton X-100 and 10% goat serum for 60 minutes at room temperature. Primary antibody was incubated overnight at 4°C. Slides were washed in PBS followed by incubation of secondary antibodies for 60 minutes at room temperature. After washing in PBS, slides were nuclear stained with ToPro-3 (Invitrogen, Carlsbad, CA) and coverslip with Shandon-Mount (Thermo Fisher Scientific, Pittsburgh, PA) before being visualized on the Zeiss LSM 510 META Confocal Laser Scanning Microscope (Carl Zeiss International, Jena, Germany). Measurement of SMI-32 positive signal area within the ROIs encompassing the VLWM was done using the public domain NIH Image program ImageJ.

### Statistical analysis

All statistical analysis was done using Prism 5.0 (GraphPad Software, Inc. La Jolla, CA). *In vivo *experiments for clinical scoring and DTI imaging were performed with n = 5 animals per treatment group, and 3 mice per group were further included in the histological studies. Data obtained from clinical severity scores and SMI-32 staining was analyzed via Two-way ANOVA. Highest severity score by disease phase and RA, λ⊥, λ|| map histograms were analyzed by one-way ANOVA. All analyses were followed by Bonferroni post-hoc test. Correlation analysis was done between λ|| and area-under-curve (AUC) of the clinical score. For all statistical analysis a p < 0.05 was considered significant.

## Results

### CXCR7 antagonism promotes complete clinical recovery during EAE

**Figure 1:**
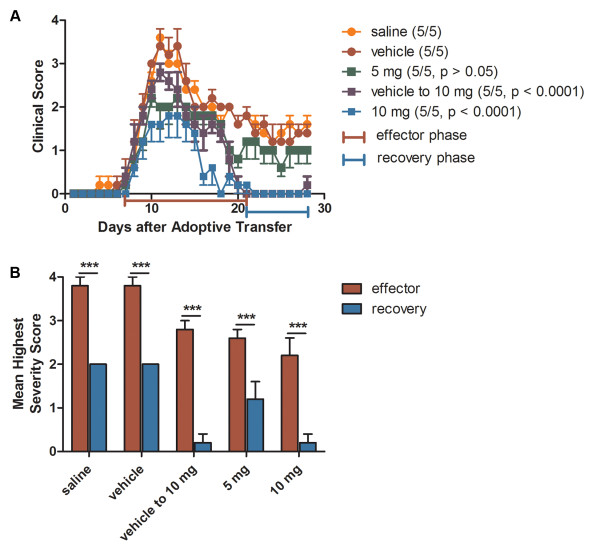
**CXCR7 antagonism ameliorates the clinical severity of EAE**. Dose response effects of the CXCR7 antagonist, CCX771, were evaluated during induction of and after treatment of ongoing adoptive transfer EAE. (**A**) Animals were grouped into those receiving daily treatment with saline (yellow), vehicle (red) or CCX771 at 5 mg/kg (green) or 10 mg/kg (blue) beginning at the time of adoptive transfer or when animals reached a score of 1 (purple). Results are expressed as mean clinical score ± SEM, n = 5. Two-way ANOVA for all treatment groups showed a strong interaction of treatment and days post-adoptive transfer with disease progression: interaction: F = 2.201, P < 0.0001; antagonist treatment: F = 93.85, P < 0.0001; days post-adoptive transfer: F = 59.06, P < 0.0001. (**B**) Comparison between the mean highest severity scores of effector phase (red bracket on A) and recovery phase (blue bracket on A). Results are expressed as mean highest severity score ± SEM. Two-way ANOVA P values summary: interaction: F = 1.71, P = 0.1659; disease phase: F = 164.57, P < 0.0001; treatment: F = 22.21, P < 0.0001).

We recently demonstrated that CXCR7 antagonism ameliorates EAE induced by adoptive transfer of encephalitogenic Th1 cells by decreasing both leukocyte entry into the CNS parenchyma and demyelination [12]. Because both loss of myelin and interactions between immune and neuronal cells lead to axonal damage [31,32], we wondered whether CXCR7 antagonism might affect the extent of recovery. Mice underwent adoptive transfer of MOG-reactive Th1 cells and were then administered daily injections of saline, vehicle, 5 or 10 mg/kg of CCX771, a specific antagonist of CXCR7 [12], and monitored for disease progression for a period of 28 days. In order to examine whether limiting inflammatory infiltrates during ongoing disease impacts on recovery, half of the vehicle-treated mice began administration of CCX771 when they attained a clinical score of 1. Consistent with our previous report [12], CXCR7 antagonism led to a dose dependent decrease in the peak severity of EAE even in mice with established disease (Figure 1A). Evaluation of disease severity during the recovery phase, 21 to 28 days post-transfer (blue bracket in Figure 1A), revealed that CCX771-treated mice exhibited a dose-dependent increase in recovery with significantly lower clinical severity scores than vehicle- or saline-treated control groups (Tables 1 and 2 and Figure 1B). All animals from both groups exhibited a highest severity score of 2 during the recovery phase, 21 to 28 days post-transfer (Figure 1B, recovery phase for vehicle and saline SEM = 0). Of interest, animals treated with CCX771 that attained a clinical score of 1 exhibited the same mean severity score upon recovery phase, 21 to 28 days post-transfer (blue bracket in Figure 1A), as those that began treatment at the time of adoptive transfer, despite a significant difference in their peak clinical disease severities during effector phase, 7 to 21 days post-transfer (red bracket in Figure 1A). (Figure 1A Two-way ANOVA interaction: F = 2.20, DFn = 108, DFd = 560, P < 0.0001; antagonist treatment: F = 93.85, DFn = 4, DFd = 560, P < 0.0001; day post-adoptive transfer: F = 59.06, DFn = 27, DFd = 560, P < 0.0001; Figure 1B Two-way ANOVA interaction: F = 1.71, P = 0.1659; disease phase: F = 164.57, P < 0.0001; treatment: F = 22.21, P < 0.0001 and Table 1: One-way ANOVA effector phase: F = 8.250, P > 0.0004; Table 2: One-way ANOVA recovery phase: F = 18.45, P < 0.0001). These data suggest that CXCR7 antagonism not only modifies disease severity during EAE but enhances overall recovery regardless of initial impairments, as accessed via clinical scoring.

**Table 1:** One-way ANOVA for effector phase.

	saline	vehicle	5 mg	vehicle to 10 mg	10 mg
**saline**	**--**	ns	*	ns	**

**vehicle**	ns	**--**	*	ns	**

**5 mg**	*	*	**--**	ns	ns

**vehicle to 10 mg**	ns	ns	ns	**--**	ns

**10 mg**	**	**	ns	ns	**--**

Summary of *p *values comparing the highest severity scores among treatment groups. ns = non-significant, * = p < 0.05, ** p < 0.01, P = 0.0004, F = 8.250, R^2 ^= 0.6226.

**Table 2:** One-way ANOVA for recovery phase.

	saline	vehicle	5 mg	vehicle to 10 mg	10 mg
**saline**	**--**	ns	ns	***	***

**vehicle**	ns	**--**	ns	***	***

**5 mg**	ns	ns	**--**	*	*

**vehicle to 10 mg**	***	***	*	**--**	ns

**10 mg**	***	***	*	ns	**--**

Summary of *p *values comparing the highest severity scores among treatment groups. ns = non-significant,* = p < 0.05, *** p < 0.001, P < 0.0001, F = 18.45, R^2 ^= 0.7868.

### DTI reveals treatment efficacy of CXCR7 antagonist on EAE mice

**Figure 2:**
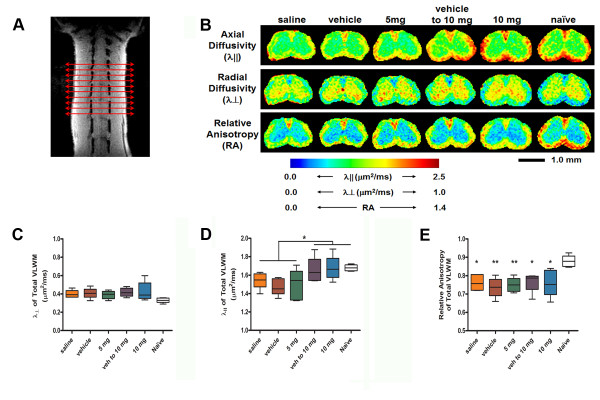
**DTI analysis shows changes in ventral white matter**. At the end of clinical assessment mice from all treatment groups and naïve littermates underwent *in vivo *DTI analysis. Spinal cord level was localized by axial scout images followed by multiple transverse slices (red arrows) to include the whole lumbar enlargement (**A**, slice thickness = 1.0 mm, field of view = 1 cm × 1 cm). Diffusion-sensitizing gradients were applied in six orientations: (Gx, Gy, Gz) = (1, 1, 0), (1, 0, 1), (0, 1, 1), (-1, 1, 0), (0, -1,1), and (1, 0, -1) with a gradient strength = 9 G/cm, duration (*δ*) = 7 ms, and separation (Δ) = 18 ms, to obtain *b *values of 0 and 0.750 s/mm^2^. Regions of interest (ROIs) encompassing the ventrolateral white matter (VLWM) was drawn manually on the DTI parameter maps (**B**). The boundary between white matter and gray matter was identified on relative anisotropy (RA) maps. The clear gray-white matter contrast was seen in RA maps of all study groups. Radial (λ⊥) and axial (λ||) diffusivities showed heterogeneous abnormalities within the VLWM, being more severe in saline- and vehicle-treated groups. Analysis of radial diffusivity and relative anisotropy do not show differences between treatment groups, suggesting no differences in myelin integrity (**C and E**, One-way ANOVA F = 3.232, P = 0.0227 and F = 5.272, P = 0.0021, respectively). Meanwhile, analysis of axial diffusivity shows a similarity between 10 mg/kg CCX771-treated mice with naïve (**D**, One-way ANOVA, F = 3.232, P = 0.0227). Results are expressed as mean of λ⊥, λ|| or RA ± SD).

*In vivo* DTI was performed to assess microstructural changes within multiple transverse slices of the lumbar enlargement of mouse spinal cords (Figure 2A) in CCX771-, vehicle- or saline-treated mice after recovery from peak EAE, plus age-matched naïve control mice. Relative anisotropy (RA) maps were generated within the manually defined regions of interest (ROIs) of VLWM (Figure 2B). Axon and myelin injury in VLWM, most severe in the control groups, was apparent in EAE mice as evidenced by the intensity changes in radial and axial diffusivity maps (Figure 2B). Statistical analysis of changes in radial diffusivity failed to show significant differences in the VLWM among study groups, suggesting no differences in myelin integrity (Figure 2C, One-way ANOVA F = 1.696, P = 0.1740). In contrast, analysis of changes in VLWM axial diffusivity detected significant decreases in groups of mice that received low dose CCX771 (5 mg/kg), vehicle or saline versus those that received high dose (10 mg/kg) and naïve mice (Figure 2D). Axial diffusivity of 10 mg/kg CCX771-treated mice resembled the values of the naïve group, while 5 mg/kg CCX771-treated mice resembled those obtained from control groups (Figure 2D, One-way ANOVA F = 3.232, P = 0.0227). Lastly, RA of VLWM showed no difference between vehicle- or saline-treated mice and CCX771-treated mice (Figure 2E, One-way ANOVA F = 5.272, P = 0.0021).

**Figure 3:**
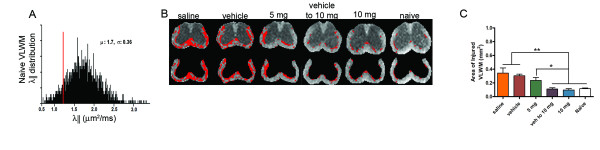
**Axial diffusivity threshold segmentation on VLWM of mouse spinal cord reveals similarities of CCX771-treated mice with naïve**. VLWM λ|| distribution from naïve spinal cord (μ = 1.7, σ = 0.36, n = 5) (**A**). Red line represents threshold of injured white matter from naïve spinal cords used as control. The background image is the λ|| maps (**B**). The red masks represent axial diffusivity threshold defined injured axon (or white matter). Saline- and vehicle-treated mice showed extensive VLWM having abnormal axial diffusivity. Statistical analysis confirmed no significant difference between naïve and 10 mg/kg CCX771-treated mice in contrast to the other groups (**C**, results are expressed as mean ± SD. One-way ANOVA, F = 7.855, P = 0.0002).

The extent of axonal preservation was assessed according to the axial diffusivity distribution [16] from the naïve spinal cords (Figure 3A) to distinguish the injured from the normal appearing VLWM (Figure 3B). No differences in the extent of injured VLWM were detected between 10 mg/kg CCX771-treated and naïve mice while 5 mg/kg CCX771-, vehicle-, and saline-treated mice exhibited gradations of injury within the VLWM (Figure 3C).

**Figure 4:**
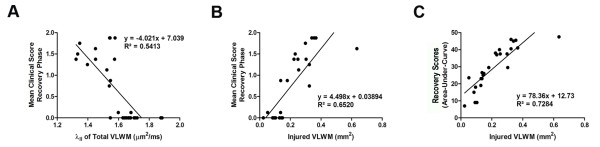
**Axial diffusivity defined abnormal VLWM volume correlates with clinical severity**. Linear regression analysis of mean clinical score during recovery phase as a function of axial diffusivity (A) and injured VLWM (B) (**A**, R^2 ^= 0.5413, F = 33.04, DFn, DFd = 1.0, 28.0, P < 0.0001; **B**, R^2 ^= 0.6520, F = 43.10, DFn, DFd = 1.0, 23.0, P < 0.0001). Area-under-curve (AUC) as a function of injured VLWM showed an R^2 ^value of 0.7284 (**C**, F = 61.69, DFn, DFd = 1.0, 23.0, P < 0.0001).

We next inquired if changes in axial diffusivity after CCX771 treatment correlated with disease recovery. To answer this question we evaluated the mean clinical score only during the recovery phase, 21 to 28 day post-adoptive transfer, as a function of axial diffusivity VLWM and injured VLWM. Both analyses showed a statistically significant linear correlation of clinical score during the recovery phase with axial diffusivity (Figure 4A, R^2 ^= 0.5413, F = 33.04, DFn, DFd = 1.0, 28.0, P < 0.0001) and injured VLWM (Figure 4B, R^2 ^= 0.6520, F = 43.10, DFn, DFd = 1.0, 23.0, P < 0.0001). In addition, linear correlation between the temporal-cumulative clinical score and injured VLWM at the time of DTI evaluation was significant (Figure 4C, R^2 ^= 0.7284, F = 61.69, DFn, DFd = 1.0, 23.0, P < 0.0001). These data strongly support a role for CXCR7 as a disease-modifying molecule during the recovery phase of EAE via axonal preservation.

### CXCR7 antagonism does not alter myelin levels during recovery from EAE

**Figure 5:**
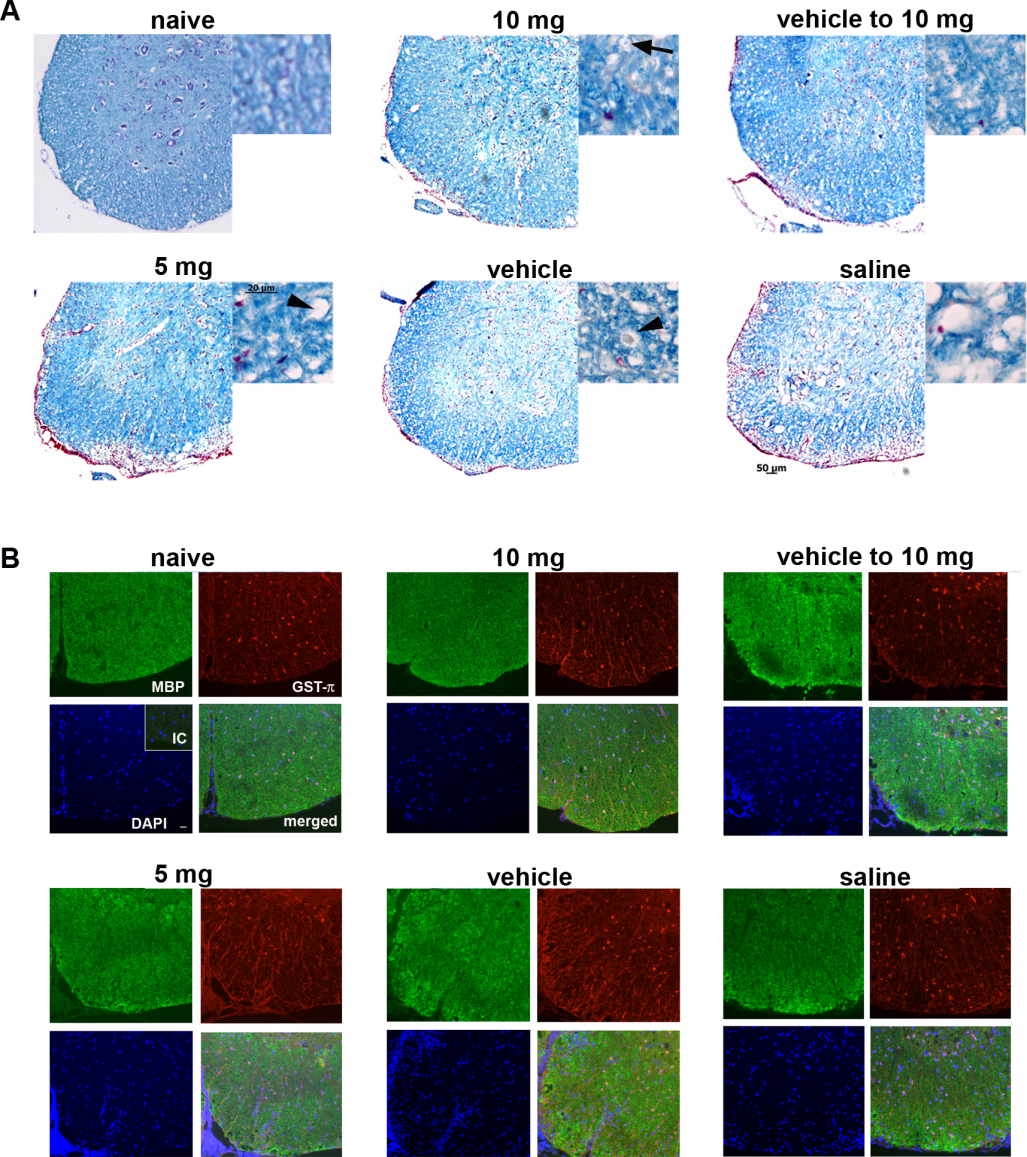
**Myelin staining is consistent with DTI findings**. Lumbar segments sections of 6 μm were stained with LFB and exhibited comparable levels of myelination around ventral horns when observed at 5X of magnification (**A **scale bar = 50 μm). Persistent inflammation at the meningeal surface was detected in 5 mg/kg CCX771-, vehicle- and saline-treated mice. At 40X magnification (offsets, scale bar = 20 μm) of the same lumbar segment shown similar myelin distribution for 10 mg/kg CCX771-treated mice than naïve. At 40X magnification (offsets, scale bar = 25 μm) normal axons could be identified (arrow) in mice treated with 10 mg/kg CCX771 while axonal bundles in different stages of degeneration were observable to a lesser extent in 5 mg/kg CCX771-treated mice but, more abundant and severe in vehicle- and saline-treated mice (arrowheads). Immunofluorescent detection of MBP (green) and GST-π (red) showed similar levels of myelin content and presence of oligodendrocytes in all treatment groups (**B **scale bar = 25 μm). As observed with LFB, DAPI nuclear staining (blue) is more evident in the 5 mg/kg CCX771-, vehicle- and saline-treated mice.

Prior studies evaluating the effect of CXCR7 antagonism during EAE revealed decreased demyelination in mice treated with CCX771 compared with vehicle-treated animals at the peak of disease [12]. Because we did not observe any differences in measurements of radial diffusivity between treatment groups after disease recovery, we performed luxol fast blue (LFB) staining of spinal cords at lumbar segments L2/3. Consistent with our DTI data, there were no apparent differences between any treatment groups in the VLWM myelin density as detected by this method (Figure 5A). Of interest, mice treated with 10 mg/kg CCX771 exhibited myelin-density staining that was comparable to naïve animals (Figure 5A, upper panels). After 28 days post-adoptive transfer, persistent inflammation was still detectable within the VLWM spinal cords of saline- and vehicle-treated mice and, to some extent, of mice treated with 5 mg/kg CCX771, but not in mice treated with 10 mg/kg CCX771 (Figure 5A, lower panels versus middle and right upper panels). We also observed pathological features consistent with hypertrophic and degenerating axonal bundles, as reported in human and rodent models of CNS degeneration and trauma (Figure 5A, lower panels, arrowhead) [33-39]. However, these features were absent in the naïve and 10 mg/kg CCX771-treated mice, where more normally appearing axons were present (Figure 5A, mid-upper panel, arrow).

To better assess myelin protein expression and oligodendrocyte numbers, we examined myelin basic protein (MBP) and GST-π+ immunoreactivity. MBP expression is frequently used for immunohistochemical analysis of demyelination and remyelination in EAE [40-43] while GST-π is a marker for mature oligodendrocytes [44-49]. Consistent with the LFB staining, MBP expression and GST-π+ immunoreactivity within the VLWM was comparable among all treatment groups. Of note, MBP staining exhibited more heterogeneity within the spinal cords of mice with EAE that did not receive higher doses of CXCR7 antagonist. These findings suggest that CXCR7 antagonism preserves axons independent of myelin recovery.

### CXCR7 antagonism ameliorates persistent CNS inflammation

**Figure 6:**
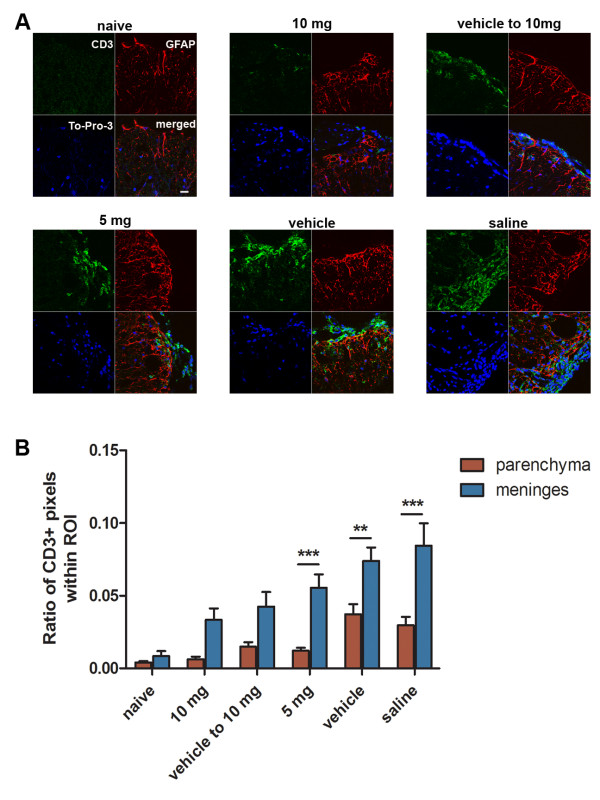
**Injured VLWM shows evidence of persistent inflammation**. Double immunofluorescence analysis of infiltrated CD3+ T-cells (green) using GFAP (red) antibody to delineate CNS parenchyma within spinal cords of mice evaluated by DTI (**A**). T cells were absent on spinal cords from naïve mice while some CD3+ lymphocytes were detected on CCX771-treated mice. However, these CD3+ T-cells were mostly restricted to meningeal spaces and within GFAP+ parenchyma. Vehicle- and saline-treated mice showed a higher infiltration of CD3+ lymphocytes within GFAP+ parenchyma. As observed with LFB, To-Pro-3 nuclear staining (blue) is more evident in the 5 mg/kg CCX771-, vehicle- and saline-treated mice. (**A **63X magnification, scale bar = 25 μm). Statistical analysis shows significant differences in CD3+ pixel ratios within ROI's (parenchyma and meninges) among treatment groups (**B **results are expressed as mean CD3+ pixels per ROI ± SEM. Two-way ANOVA P values summary: interaction F = 2.49, P = 0.0347; antagonist treatment F = 13.52, P < 0.0001; area of CNS (parenchyma or meninges) F = 52.86, P < 0.0001).

To assess the phenotype of immune cells that persist within the CNS parenchyma during recovery, spinal cord tissues from our five treatment groups and naïve controls were evaluated for meningeal and parenchymal CD3+ cells in conjunction with GFAP expression, the latter to delineate meningeal and parenchymal borders. All treatment groups exhibited meningeal CD3+ cells, which were increased in numbers in vehicle- and saline-treated mice compared with mice treated with 5 mg/kg or 10 mg/kg CCX771 (Figure 6A). Parenchymal infiltration of CD3+ cells was also increased in vehicle- and saline-treated mice compared with mice treated with CCX771, which exhibited few parenchymal CD3+ cells (Figure 6A). Quantitative analysis of meningeal and parenchymal CD3+ staining within both the parenchyma and meninges of mice in each treatment group revealed significant differences between the two CNS regions in mice treated with low dose CCX771 (5 mg/kg) and in vehicle- or saline-treated controls but not in mice treated with high dose CCX771 (10 mg/kg) (Figure 6B, Two-way ANOVA interaction: F = 2.49, DFn = 5, DFd = 118, P = 0.0.347; antagonist treatment: F = 13.52, DFn = 5, DFd = 118, P < 0.0001; area of CNS (parenchyma or meninges): F = 52.86 DFn = 1 DFd = 118, P < 0.0001). Comparison of CD3 staining within the spinal cord parenchyma, but not the meninges, across treatment groups revealed highly significant differences between all treatment groups and vehicle-treated controls (Table 3 One-way ANOVA parenchyma: F = 10.56, p < 0.0001). These data indicate that CXCR7 antagonism prevents persistent T cell infiltration within the spinal cord parenchyma, which could potentially contribute to ongoing axonal damage.

**Table 3:** One-way ANOVA comparing CD3+ pixels ratio within parenchyma.

	10 mg	vehicle to 10 mg	5 mg	vehicle	saline
**10 mg**	**--**	ns	ns	***	**

**vehicle to 10 mg**	ns	**--**	ns	**	ns

**5 mg**	ns	ns	**--**	***	ns

**vehicle**	***	**	***	**--**	ns

**saline**	**	ns	ns	ns	**--**

Summary of *p *values comparing the ratio of CD3+ pixels within parenchyma from each treatment group. ns = non-significant, * = p < 0.05, ** p < 0.01, *** p < 0.001, P < 0.0001, F = 10.56, R^2 ^= 0.4599.

### CXCR7 antagonism limits axonal injury during EAE

**Figure 7:**
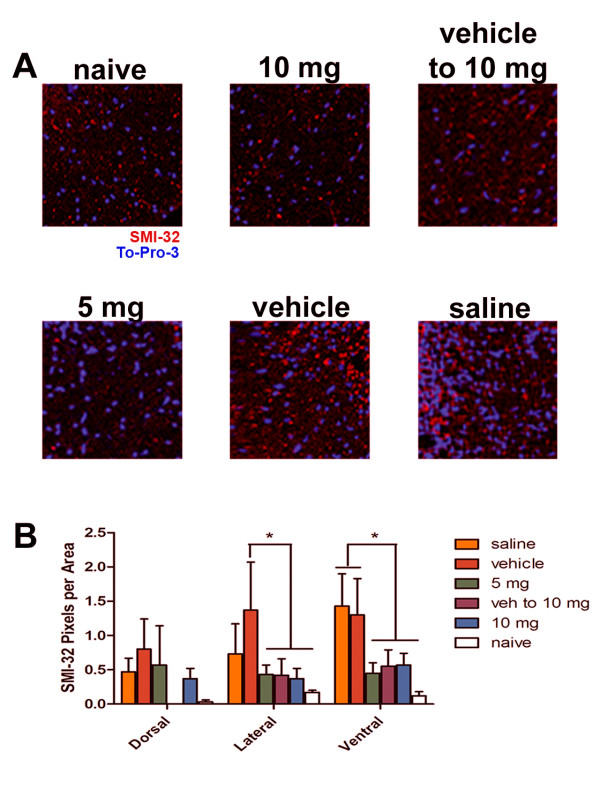
**Injured VLWM shows abnormal dephosphorylation of Neurofilament H**. Immunofluorescence analysis of NF-H was assessed using SMI-32 antibody immunoreactivity to detect dephosphorylation within spinal cords after DTI evaluation (**A**). Higher SMI-32 staining within VLWM was observed in saline- and vehicle-treated mice in contrast to naïve controls, while CCX771-treated mice were more comparable to control mice (63X magnification, scale bar = 25 μm). Statistical analysis shows significant differences within lateral and ventral portions of the spinal cord white matter area (**B **results are expressed as mean SMI-32+ pixels per area ± SEM. Two-way ANOVA P values summary: interaction F = 0.3671, P = 0.9568; antagonist treatment F = 3.248, P = 0.0107; area F = 1.4661, P = 0.2382.).

In order to assess the contents of spared spinal cord VLWM in treatment groups after recovery from EAE, spinal cords sections encompassing L2/3, which were the same segments used for LFB staining, were examined for the extent of neurofilament H (NF-H) dephosphorylation via anti-SMI-32 immunofluorescence, as previously reported [16,17,50,51]. SMI-32 immunopositivity was found predominantly in neuronal cell bodies with little detectable staining in the white matter in both groups of 10 mg/kg CCX771-treated mice and in naïve animals (Figure 7A, upper panels). A similar pattern was observed in the 5 mg/kg CCX771-treated mice group (Figure 6A, left lower panel). Extensive SMI-32+ immunoreactivity, however, was detected in the spinal cord VLWMs of either saline- or vehicle-treated mice, even in areas where myelin was present (Figure 5, middle and right lower panels), consistent with DTI findings of axonal damage (Figure 7A, middle and right lower panels). Further statistical analyses of SMI-32+ pixels/area within dorsal, lateral and ventral spinal cord white matter of mice in each treatment group revealed significant decreases between CCX771-treated and naïve versus control animals only within lateral and ventral regions (Figure 7B, 2-way ANOVA interaction: F = 0.37, DFn = 10, DFd = 71, P = 0.9568; antagonist treatment: F = 3.25, DFn = 5, DFd = 71, P = 0.0107; white matter area: F = 1.46 DFn = 2, DFd = 71, P = 0.2388). These data support the DTI findings and that CXCR7 antagonism in the setting of EAE prevents axonal damage in a dose dependent fashion.

## Conclusions

The current study utilized *in vivo *DTI to evaluate the role of CXCR7 in recovery from EAE and provides compelling evidence that CXCR7 antagonism prevents inflammatory-mediated injury to axons. Axial diffusivity measurements of injured spinal cord VLWM in mice treated with CXCR7 antagonist were undistinguishable from those of naïve mice, which were both significantly different from vehicle- and saline-treated mice. Consistent with this, extensive SMI-32+ immunoreactivity within spinal cord VLWM was detected in both saline- or vehicle-treated mice but not in tissues obtained from mice treated with CXCR7 antagonist. In contrast, there were no differences in radial diffusivity measurements or extent of LFB staining, MBP+ and GST-π+ immunoreactivity between groups, suggesting that axonal preservation in the setting of CXCR7 antagonism may be independent of myelin repair. A linear correlation between injured VLWM and mean maximal disease severity scores was observed only during recovery phase, consistent with the notion that immune-mediated axonal damage persists at late stages of EAE in control-treated animals. Consistent with this, we observed significant increases in persistent inflammatory infiltrates within the parenchyma of saline- and vehicle-treated mice that was abrogated with CCX771 administration. These control tissues also exhibited the presence of pathological features consistent with hypertrophic-degenerative axonal bundles [33-39], suggesting that axonal damage and not lack of myelin is the key pathology in mice with chronic deficits. These findings support the relevance of CXCR7 antagonist treatment to preserve axonal integrity and indicate that DTI may be used to determine the treatment effects of small molecule inhibitors on pathological biomarkers of CNS autoimmunity.

A growing body of evidence supports the notion that neurologic dysfunction in MS occurs as a result of axonal degeneration [1], which may be dependent on or occur independently of chronic demyelination [52]. Demyelination-dependent mechanisms include ongoing axonal toxicity due to the up-regulation of sodium channels throughout the length of demyelinated axons in MS and EAE. Consistent with this, mice deficient in Na+ channel beta2 subunits exhibit decreased axonal degeneration and loss during EAE compared with wild-type animals, despite similar levels of CNS inflammation [53]. However, myelinated axonal segments continuously exposed to activated macrophages or their products can undergo focal degeneration with associated mitochondrial dysfunction [8], suggesting that inflammatory mediators may induce axonal damage without demyelination [8]. Consistent with this, using the Biozzi ABH murine model, Jackson and colleagues demonstrated that axonal pathology is the primary event in relapsing-progressive EAE [54]. These investigators observed axonal loss in the acute phase of disease prior to demyelination with further axonal loss in the absence of chronic inflammation during relapses. In our study, vehicle-treated mice with ongoing disease could attain complete recovery upon initiation of CXCR7 antagonist, suggesting peak disease contains a reversible component. Because all groups exhibited equal levels of radial but not axial diffusivity, this reversibility may be attributed to axonal damage. In our previous work, we demonstrated that CXCR7 antagonism decreased infiltration of immune cells to CNS parenchyma as assessed by both, immunohistochemistry and flow cytometry [12]. Thus, T cell entry is likely to be a primary event that reversibly injures axons. Consistent with this, CD8+ T cells reportedly cause bystander axonal damage in murine cerebellar slice cultures [55]. Another putative mechanism is T cell-mediated axonal dysfunction via microtubule destabilization, which was observed to cause synaptic impairment and accumulation of APP aggregates [56,57]. Further studies utilizing CXCR7 antagonist are ongoing to address these possible mechanisms.

Recent studies indicate that oligodendrocyte precursor cells (OPCs) express both CXCL12 receptors and that CXCL12 is essential for remyelination [58-62]. Thus, it is possible that CXCR7 antagonism impacts on OPC biology. Although EAE-recovered vehicle- versus CXCR7 antagonist-treated mice exhibit equivalent levels of myelin, as assessed by DTI, LFB staining, MBP and GST-π immunohistochemal analyses, detection of new myelin may be better accomplished using non-immune-mediated models of demyelination, such as cuprizone toxicity. Mice fed the copper chelator cuprizone display specific demyelination of the corpus callosum, which exhibits remyelination upon cessation of toxin [59]. Studies examining CXCR7 antagonism in this context may be more conducive for the evaluation of OPCs recruitment and maturation. Alternatively, as CXCR7 antagonism was previously shown to limit demyelination during peak EAE [12], it is possible that the preserved axonal integrity is due to preservation of myelin.

Prior studies have revealed a strong correlation between changes in axial diffusivity and white matter pathology via quantitative pixelwise DTI analysis [17], strengthening the usefulness of axial diffusivity as a biomarker for axonal integrity. Nevertheless, while several studies have demonstrated efficacy for DTI in evaluating *in vivo *treatment modalities in animal models of CNS injury [63,64], this is the first study evaluating a pharmacological inhibitor in a murine model of MS *in vivo*. Mi et al., showed that treatment of Sprague-Dawley rats with anti-LINGO-1 promotes recovery from MOG-induced EAE, either by intrathecal or systemic delivery [65]. These investigators also correlated EAE amelioration with axonal integrity as assessed by axial diffusivity; however, imaging was performed *post-mortem *in fixed spinal cord specimens. More recently, DTI has been used successfully in patients for the evaluation of G-CSF treatment for amyotrophic lateral sclerosis [66] and anti-tubercular treatment for *Tuberculous meningitis *[67], highlighting the feasibility of DTI for evaluating CNS lesions and inflammation.

In summary, our study provides evidence that CXCR7 antagonism in the setting of CNS autoimmunity prevents axonal loss, as assessed by both novel imaging and standard histological modalities. In addition, our work supports the use of DTI, especially axial diffusivity, as a non-invasive biomarker for the detection of axonal integrity in EAE and MS.

## Competing interests

The authors declare that they have no competing interests.

## Authors' contributions

L.C.O. and R.S.K. designed research and wrote paper; L.C.O., Y-J.C., D.D. and J.H.K. conducted research; acquisition of data, analysis and interpretation of data; S-K.S. and R.S.K., analysis and interpretation of data. All authors read and approved the final manuscript.
